# Comparison of two independent systematic reviews of trials of recombinant human bone morphogenetic protein-2 (rhBMP-2): the Yale Open Data Access Medtronic Project

**DOI:** 10.1186/s13643-017-0422-x

**Published:** 2017-02-15

**Authors:** Jeffrey Low, Joseph S. Ross, Jessica D. Ritchie, Cary P. Gross, Richard Lehman, Haiqun Lin, Rongwei Fu, Lesley A. Stewart, Harlan M. Krumholz

**Affiliations:** 1 Department of Internal Medicine, Yale School of Medicine, 333 Cedar Street, New Haven, CT 06510 USA; 2 Center for Outcomes Research and Evaluation, Yale-New Haven Hospital, 1 Church Street, Suite 200, New Haven, CT 06510 USA; 3 Section of General Internal Medicine, Department of Internal Medicine, Yale School of Medicine, P.O. Box 208093, New Haven, CT 06510 USA; 4 Robert Wood Johnson Foundation Clinical Scholars Program, P.O. Box 208088, New Haven, CT 06510 USA; 5 Department of Health Policy and Management, Yale School of Public Health, P.O. Box 208034, New Haven, CT 06520 USA; 6 Department of Primary Care Health Sciences, Oxford University, 33 St Ebbes Street, Oxford, OX1 1PU UK; 7 Department of Biostatistics, Yale School of Public Health, P.O. Box 208034, New Haven, CT 06520 USA; 8 Department of Public Health and Preventive Medicine, Pacific Northwest Evidence-based Practice Center, Oregon Health & Science University, 3181 S.W. Sam Jackson Park Road, Portland, OR 97239-3098 USA; 9 Centre for Reviews and Dissemination, University of York, Heslington, York, YO10 5DD UK; 10 Section of Cardiovascular Medicine, Department of Internal Medicine, Yale School of Medicine, P.O. Box 208093, New Haven, CT 06510 USA

**Keywords:** Data sharing, Data interpretation, Systematic review, Meta-analysis

## Abstract

**Background:**

It is uncertain whether the replication of systematic reviews, particularly those with the same objectives and resources, would employ similar methods and/or arrive at identical findings. We compared the results and conclusions of two concurrent systematic reviews undertaken by two independent research teams provided with the same objectives, resources, and individual participant-level data.

**Methods:**

Two centers in the USA and UK were each provided with participant-level data on 17 multi-site clinical trials of recombinant human bone morphogenetic protein-2 (rhBMP-2). The teams were blinded to each other’s methods and findings until after publication. We conducted a retrospective structured comparison of the results of the two systematic reviews. The main outcome measures included (1) trial inclusion criteria; (2) statistical methods; (3) summary efficacy and risk estimates; and (4) conclusions.

**Results:**

The two research teams’ meta-analyses inclusion criteria were broadly similar but differed slightly in trial inclusion and research methodology. They obtained similar results in summary estimates of most clinical outcomes and adverse events. Center A incorporated all trials into summary estimates of efficacy and harms, while Center B concentrated on analyses stratified by surgical approach. Center A found a statistically significant, but small, benefit whereas Center B reported no advantage. In the analysis of harms, neither showed an increased cancer risk at 48 months, although Center B reported a significant increase at 24 months. Conclusions reflected these differences in summary estimates of benefit balanced with small but potentially important risk of harm.

**Conclusions:**

Two independent groups given the same research objectives, data, resources, funding, and time produced broad general agreement but differed in several areas. These differences, the importance of which is debatable, indicate the value of the availability of data to allow for more than a single approach and a single interpretation of the data.

**Systematic review registration:**

PROSPERO CRD42012002040 and CRD42012001907.

## Background

Systematic reviews and meta-analyses [1] based on individual participant-level data (IPD) from randomized controlled trials (RCTs) are considered to provide the highest level of rigor for evaluating the evidence for a clinical question [2]. Such reviews offer the possibility of using hierarchical statistical techniques that better handle sources of heterogeneity, allow for sub-group analyses, and facilitate assessment of rare events. Previously, IPD meta-analyses have modified [3–8] or overturned [9] the results of previous meta-analyses based on the published literature alone.

Efficient and unbiased mechanisms to replicate research findings are essential for maintaining high levels of scientific credibility [10]. The premise of replication efforts is that different groups, employing rigorous methods, may take different approaches and come to different conclusions on a previously addressed question. Recent efforts to promote data sharing by the National Institutes of Health, [11, 12] the pharmaceutical industry, [13, 14] and partnerships between academia and industry [15, 16] have made replication an increasingly available mechanism to test the validity of clinical trial conclusions. This work is particularly important for systematic reviews and meta-analyses, which frequently form the basis of professional society and government guideline recommendations [17].

Previous studies have sought to determine whether systematic reviews are replicable, with new teams performing new searches, summaries, and analyses of the literature for a particular question. These studies, which compare systematic reviews of the published literature conducted at different time points, suggested that groups investigating the same research question may differ in their findings [18–21], though most often, these differences were attributed to search strategy [6, 19, 22–25]. However, it is uncertain if replication of meta-analyses, particularly those with the same research objectives, participant-level data, time, and funding, would employ the same analytic methods or arrive at identical findings. A thorough understanding of the reliability of meta-analysis requires an empiric assessment of how two distinct teams of investigators would employ meta-analytic techniques to address the same clinical question. Accordingly, we sought to determine if two independent centers, each of which were contracted to pursue identical research questions concurrently, with access to identical IPD, would employ identical methods in the areas of data use and statistical analysis and report identical, or at least consistent, results and conclusions.

## Methods

### Study design

We retrospectively compared the research methods and results of the final comprehensive publications of two meta-analyses performed in the context of full systematic reviews of recombinant human bone morphogenetic protein-2 (rhBMP-2) prepared by two independent centers, Center A [26, 27] from the University of York and Center B [28, 29] from Oregon Health & Science University, and focused on (1) meta-analysis trial inclusion criteria; (2) statistical methods; (3) summary risk estimates; and (4) conclusions.

Trial inclusion criteria were defined as study characteristics necessary for inclusion in meta-analysis. We explicitly compared, for primary and secondary endpoint meta-analyses, as well as safety analyses, the trials used by both centers for each analysis. For methods, we compared centers’ reported outcomes at various time points as well as statistical methods. We compared the centers’ risk estimates for all primary outcomes for efficacy as well as safety at all time points. In consideration of these factors, we provide a subjective comparison of the overall conclusions drawn by each center.

### Conducting the systematic reviews and IPD meta-analysis

**Fig. 1:**
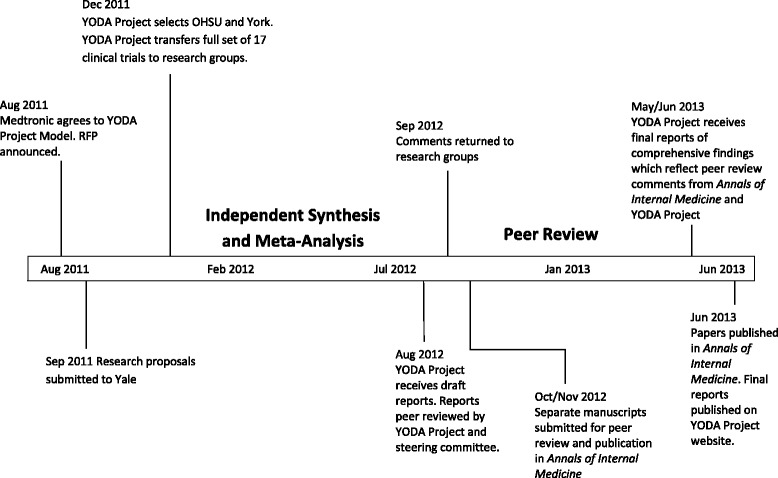
YODA Project timeline for the independent synthesis and meta-analysis of rhBMP-2 clinical trials, including their publication

Following controversy in the literature surrounding adverse events related to rhBMP-2 including cancer, in August 2011, Medtronic agreed to participate in the Yale University Open Data Access (YODA) Project model, which has been described previously (Fig. 1) [30]. Appendix 1 provides additional context on the particular clinical controversy covered by these reviews. Our analysis will focus on systematic review reproducibility rather than this particular clinical question which has already been well described in the literature. An open request for proposal was announced by the YODA Project to solicit applications from external investigators with preliminary research aims to study the safety and efficacy of rhBMP-2. The YODA Project selected research groups from Oregon Health & Science University (OHSU) and the University of York in the UK (York). These leading centers specialize in the conduct of systematic reviews and bring internationally recognized primary investigators who have made significant contributions to methodology development for organizations including the Cochrane Collaboration and the Agency for Healthcare Research and Quality (AHRQ). Based on feedback from OHSU and York, a set of reconciled aims were developed to ensure a common scope (Table 1) [31]. Each group independently developed its protocol for conducting the systematic review and deposited the full protocol with the YODA Project. Both groups registered short versions of their protocols without detailed methods for analysis on the PROSPERO registry of systematic reviews on February 23, 2012 (CRD42012002040 and CRD42012001907).

The YODA Project transferred the full set of Medtronic data relating to rhBMP-2 to the centers in early December 2011. This included full de-identified individual participant-level data for 17 trials, consisting of 8 pilot studies, 8 pivotal RCTs, and 1 study terminated for commercial reasons. The total number of participants was 2091, consisting of 1077 rhBMP-2 recipients and 1014 control participants. Also included were protocols, data dictionaries, internal reports consisting of summaries of study data, and brief adverse event case histories. In addition, 1229 MedWatch adverse event reports submitted to the US Food and Drug Administration between July 2003 and July 2012 were provided.

Each center completed IPD meta-analyses on the effectiveness and harms of rhBMP-2 in the context of full systematic reviews. Each site was responsible for determining the appropriateness of conducting a systematic review as well as its methods and research questions within the scope of the specified research aims. The project was designed so the review groups would work in parallel and have no mutual communication about their approaches. Questions from the groups were communicated through the YODA Project review coordinator so that there was no direct communication between the groups and Medtronic.

Draft reports of comprehensive findings were received from both groups by the YODA Project in mid-August 2012. These reports were peer-reviewed by separate review teams consisting of members of the YODA Project and steering committee, which included clinical, statistical, and methodological experts, as well as by a representative from Medtronic. A peer reviewer had access to only one of the two reports at any time before final publication, and there was no communication between the separate review teams. Comments were returned to the research groups in September 2012. The groups prepared separate manuscripts for submission for publication in the *Annals of Internal Medicine*. Final reports of comprehensive findings, which reflected peer review comments from the journal and from the YODA Project, were received in summer 2013. These comprehensive reports, which we review in this paper, were published on the YODA Project website congruently with the articles in the *Annals* on June 18, 2013. The data set has subsequently been made available to additional researchers through a request process [32]. The Human Investigation Committee at Yale University determined that this study is not considered to be Human Subjects Research and did not require further review.

**Table 1:** Explicit research aims provided to the 2 independent centers by the YODA Project

Research aims
1. Identify all relevant studies, across all uses and sponsor (i.e., Medtronic sponsored, others).
2. Determine the questions that were addressed by these studies.
3. Evaluate the quality of the studies. Assess the risk of bias associated with the design, conduct, and reporting of each clinical study, including those identified via the systematic review and those provided by Medtronic, and, if present, how bias may have affected assessment of effectiveness and harms.
(a) Assessment of study design and conduct should include evaluation of internal validity, methods used to ascertain outcomes and other policies and procedures for data collection, as well as the integrity of case report form adjudication.
(b) Assessment of study reporting should include selective publication and selective reporting
(c) Summary of these findings should include:
i. What conclusions can be drawn by assessing the full body of data and what gaps in knowledge remain, taking into account results from the evaluation of quality and risk of bias
ii. An assessment of applicability of these studies
4. Conduct meta-analyses from studies identified via the systematic review, if appropriate, and using patient-level data, if possible. If not appropriate there should be another approach to summarizing the data. The analysis should consider the following:
a. For effectiveness, meta-analysis should consider patient-centered outcomes (i.e., quality of life and functional status), as well as surrogate outcomes (i.e., fusion as determined by radiography).
b. For safety, meta-analysis should include local effects such as inflammation, heterotopic bone formation, pain, osteolysis and instability, and downstream or systematic effects such as leg pain and weakness, retrograde ejaculations, and possible increased risk of cancer.

## Results

### Meta-analysis inclusion criteria

Trial inclusion was largely similar with a primary difference of IPD obtained from a single published RCT. Both centers chose only to include RCTs of rhBMP-2 in spinal fusion in their meta-analysis, and both groups analyzed 11 of the RCTs. Center A obtained IPD from, and included an additional non-industry sponsored RCT by, Glassman et al. [33] for its analysis of effectiveness but excluded it when looking at harms since events were reported differently and without information on when they occurred. Though Center B identified this study, it did not solicit IPD from its authors and was able to include only a qualitative analysis.

### Research methodology

Research methodology differed primarily in the choice of stratification, with minor differences in the choice of statistical methods. For analyses of benefits, Center A included trials that compared rhBMP-2 with standard bone grafting techniques across all surgical approaches. As the primary analysis, Center A performed a standard two-stage meta-analysis along with a sub-group analysis that did not find evidence of differences between surgical approaches.

Center B stratified by surgical approach for effectiveness and most harms and determined that only two of the four surgical approaches (anterior lumbar interbody fusion (ALIF) and posterolateral fusion (PLF)), which were studied in multiple RCTs, provided adequate data for meta-analysis. Center B employed a one-stage meta-analysis, using mixed effects regression models. The study comparing rhBMP-2 with lumbar disc prosthesis was included in the analysis of cancer and death, which was not stratified by surgical approach.

Both centers studied the same primary outcomes for effectiveness and reported them at the same time points of 6 weeks and 3, 6, 12, and 24 months after surgery (Table 2).

Similar outcomes were also reported between the centers for harms up to 4 weeks and then up to 24 months for general adverse events, and up to 48 months for cancer and death.

Neither group found evidence of an rhBMP-2 dose-response relationship or heterogeneity in groups that received high-dose forms of rhBMP-2, so all dose formulations were combined.

For harms, Center A chose to combine all trials using a generalized mixed effects model since specific adverse events were few at the trial level. Center B also used a generalized mixed effects model with stratification by surgical approach, except for cancer and death.

**Table 2:** Results from meta-analyses conducted independently by Centers A and B examining measures of efficacy and safety associated with rhBMP-2

Outcome	Center	Surgical approach	No. of studies (n)	Effect size (95% CI)	Treatment advantage
ODI	A	All	12 (1368)	(0-100) -**3.48 (-6.47 to -0.49)**	BMP
	B	ALIF	5 (423)	(0-50) -**7.35 (-14.00 to -0.70)**	BMP
		PLF	4 (650)	(0-50) -1.98 (-4.86 to 0.90)	Neither
SF-36 PCS	A	All	12 (1303)	(0-100) **1.93 (0.63 to 3.22)**	BMP
	B	ALIF	5 (421)	(0-100) **3.68 (0.86 to 6.49)**	BMP
		PLF	4 (644)	(0-100) 1.10 (-0.6 to 2.86)	Neither
Back pain	A	All	12 (1326)	(0-10) -**1.58 (-2.65 to -0.51)**	BMP
	B	ALIF	4 (409)	(0-20) -**0.74 (1.49 to 0.00)**	BMP
		PLF	4 (649)	(0-20) -0.31 (-0.76 to 0.15)	Neither
Leg pain	A	All	12 (1326)	(0-10) -0.59 (-1.27 to 0.09)	Neither
	B	ALIF	4 (409)	(0-20) -0.60 (-1.28 to 0.08)	Neither
		PLF	4 (648)	(0-20) -0.34 (-0.82 to 0.13)	Neither
Fusion	A	All	10 (1078)	RR **1.14 (1.03 to 1.25)**	BMP
	B	ALIF	5 (416)	RR 1.05 (0.88 to 1.24)	Neither
		PLF	4 (637)	RR 1.16 (0.96 to 1.41)	Neither
Cancer	A	All up to 48 months	11 (1281)	RR 1.98 (0.86 to 4.54)	Neither
	B	All 24 months	5 (1450)	RR **3.45 (1.98 to 6.00)**	Control
		All 48 months	4 (1183)	RR 1.82 (0.84 to 3.95)	Neither

*CI* Confidence Interval, *ODI* Oswestry disability index. Lower favors rhBMP-2. *ALIF* Anterior lumbar interbody fusion, *PLF* Posterolateral fusion, *RR* Relative risk, *SF-36 PCS* Short Form 36 Physical Component Score. Higher favors rhBMP-2. For back and leg pain, Center A used a 0-10 scale and Center B used a 0-20 scale. Lower favors rhBMP-2. Bolded values statistically significant

### Summary results estimates

**Fig. 2:**
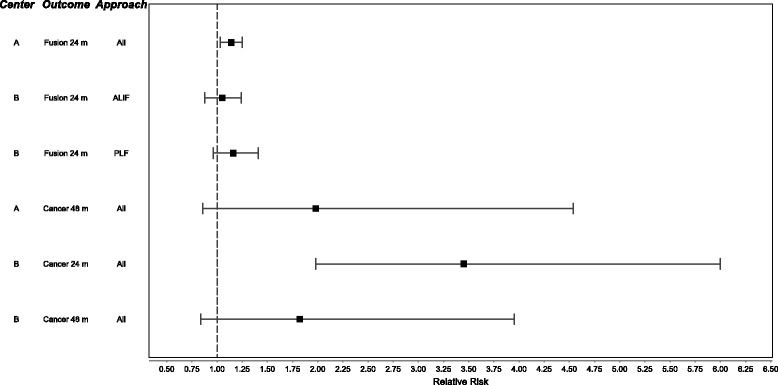
Forest plots from Center A and Center B meta-analyses examining likelihood of bone fusion and cancer risk associated with rhBMP-2

The groups obtained similar results in summary estimates of most clinical outcomes and adverse events, although there were notable differences. Center A found a statistically significant increase in fusion rate at 24 months (12% over controls) combining data across all surgical approaches. In contrast, Center B, reporting results for each surgical approach separately, did not find a significant increase in fusion at 24 months. For reducing back pain and overall disability, Center A found a statistically significant advantage for all time points from 6 months onwards when combining data from all approaches, with no statistically significant difference in the effectiveness of rhBMP-2 by surgical approach (Fig. 2). For Center B, differences for pain reduction were statistically significant from 3 months onward for ALIF, but only at the 6-month time point for PLF.

Findings were not identical for cancer; Center B reported a statistically significant increased risk at 24 months with the use of rhBMP-2, and Center A did not report at 24 months. Neither group found a significantly increased risk of cancer associated with rhBMP-2 at 48 months (Fig. 2). Both groups reported similar but not identical findings for the frequency of regular adverse events.

### Summary conclusions

Center A interpreted benefits to fusion and postoperative pain as “clinically insignificant” and increased cancer incidence as “inconclusive,” noting that “whether this increased risk is genuine is uncertain” (Table 3). Overall, by this analysis alone, rhBMP-2 seemed to offer improved rates of fusion with similar clinical outcomes compared with standard techniques at the expense of increased reports of back and leg pain in the early postoperative period.

In contrast to Center A’s report, Center B found “moderate-strength evidence of no consistent differences between rhBMP-2 and ICBG in…fusion rates.” In addition, it reported a statistically significant increase in cancer at the 24-month time point, while noting that “This finding should be interpreted with caution because cases were heterogeneous.” Overall conclusions from Center B seemed to indicate more strongly than those of Center A that rhBMP-2 had no additional clinical benefit. Center B reported that its “analysis underscores that more definitive evidence about harms was needed before rhBMP-2 became widely used” and that “On the basis of the currently available evidence, it is difficult to identify clear indications for rhBMP-2 in spinal fusion. This analysis shows almost no clinical benefit for the product while raising questions about the potential risk for cancer.”

**Table 3:** Conclusions made by Centers A and B after conducting independent summary reviews and meta-analysis of rhBMP-2 trials

Center	Conclusions
A	“At 24 months, rhBMP-2 increases fusion rates, reduces pain by a clinically insignificant amount, and increases early postsurgical pain compared with ICBG. Evidence of increased cancer incidence is inconclusive.”
	“The use of rhBMP-2 in spinal fusion surgery increases the likelihood of successful fusion at up to 24 months, but this does not seem to translate into a clinically significant reduction in pain. The small improvements in fusion and in the level of pain reduction, which manifest after 6 months, also seem to come at the expense of more frequent pain in the immediate postoperative period and, possibly, an increased number of cancer cases. We believe that it is important that clinicians explain these findings to patients so that they can make informed choices about the type of surgery they would prefer.”
B	“In spinal fusion, rhBMP-2 has no proven clinical advantage over bone graft and may be associated with important harms, making it difficult to identify clear indications for rhBMP-2.”
	In conclusion, we found…no evidence that rhBMP-2 is more effective than ICBG in spinal fusion, with some evidence of an association with important harms. More research is needed to provide more reliable estimates of risk for cancer and other adverse events and to identify patient populations in which use of rhBMP-2 may be beneficial, such as cases where use of bone graft alone is associated with a high risk for pseudarthrosis. On the basis of the currently available evidence, it is difficult to identify clear indications for rhBMP-2 in spinal fusion.”

## Discussion

In our study of two independent centers provided with identical objectives, data, resources, and time to conduct concurrent meta-analyses, we found that the centers did not report identical methods, results, and interpretations. In addition, the potential benefit of additional analyses of the same data was not limited solely to increasing confidence through replication. Separate analyses revealed nuances of differences, with potential interpretations for clinical management, which could be produced from the same data set using valid methods. These findings, even though largely similar, support the case for greater sharing and access to clinical data as a way to maximize public dialogue about the meaning of the data and to ensure that a single interpretation does not lead people to believe there is no other possible approach.

The centers took different but methodically defensible approaches in their attempts to best represent the results of this data set in a relevant and valid way. Review methods differed based on data stratification and IPD obtained from an additional trial. One group chose to combine data across all surgical approaches, finding little heterogeneity in trials by approach. The other group chose to stratify and analyze by surgical approach, forgoing increased statistical power in recognition of the real differences and adverse event concerns between different surgical approaches, and to present in a format perhaps more intuitive to spine surgeons. Study inclusion diverged, with one group obtaining IPD from an additional trial not funded by Medtronic and including it in the analysis of effectiveness. Even with the proliferation of standards in methodology, this demonstrates that we can expect some differences in how two similarly qualified groups might choose to conduct a complex systematic review. This diversity in methods has the potential to add to the depth of our understanding of a product and reinforces that additional value can be tapped from a data set with open access.

These differences in approach led to differences in summary estimates. In the case of the outcome of spinal fusion, this led to a difference that had statistical relevance even as the group discounted the clinical importance. Nevertheless, this finding could support the argument in the spine literature that the use of this product is warranted in certain indications and select cases where the risk of non-union is great and its consequences potentially disastrous [34, 35]. In contrast, this difference was no longer detectable when data were stratified by a surgical approach in the other review, and surgeons looking at these data alone might see fewer instances where this product would be beneficial. For estimates of cancer, there were slight differences in the time points reported. Center B showed a statistically significant increase in cancer at the 24-month time point but concurred that cancer was not significantly increased for longer follow-up. In both cases, the absolute risk of cancer was low, and the cancer types represented were heterogeneous.

In contrast to previous studies of concurrent meta-analyses in nutrition and endometrial cancer [7] and immunotherapy treatments for spontaneous abortions, this study found that concurrently conducted meta-analyses examining the same data arrived at conclusions that readers may or may not interpret similarly. Currently, it appears that nearly all meta-analyses are conducted by single groups, without replication. Additional analyses necessitate the sharing of data, and this in itself can bring important benefits. Information in the published literature is often incomplete. Data sharing has the potential to allow for a more complete picture of the benefits and harms of a treatment based on the totality of available evidence. Data are often collected or subsidized at the public expense and need to be made more widely available for the public benefit. Across a diverse array of fields, open access to data and the potential for reanalysis can, at the minimum, strengthen confidence in the findings of a systematic review while offering the potential to add to or even alter the conclusions about an intervention.

While there are many benefits and arguments for greater data sharing, these benefits must be considered in light of potential downsides that might come with additional analyses based on the same data. In this project, we addressed industry concerns around spurious analysis and litigation, as well as biased and methodologically flawed studies which might unfairly taint a product. Academia too faces challenges around credit, bias, and the potential for conflicting messages to confound decision-making. Ultimately, we believe a process of frameworks, like the YODA Project, and norms can help manage these potential problems and unlock the benefits that come with greater sharing of data.

The generalizability of our findings to other settings is not known. However, the design of our approach should have made it more likely that the results would have been the same rather than different. The two groups were provided with the same data from all manufacturer-sponsored studies which, for rhBMP-2, represented the vast majority of high-quality studies on this product. Studies of the other questions could be limited by differences in search strategies and disagreement over key studies. The groups also received identical funding from an outside organization, and neither the groups nor the funders had any financial interest in this product.

## Conclusions

Two independent and expert review groups that performed independent meta-analyses of rhBMP-2 came to broadly similar findings, though with some differences on the statistical significance of primary analyses of fusion and cancer. The clinical importance of the differences may be debatable, and even the authors of this article differed in their interpretations of the results and conclusions presented in these analyses. What is certain is that the methods and interpretations were not identical and had different points of emphasis. This underscores the importance of making data more openly available for the purpose of additional scientific inquiry to maximize the knowledge that can be extracted.

## Appendix 1

### rhBMP-2 background and summary findings

Clinical background: spinal fusion is a commonly performed surgery to correct spinal instability and commonly accompanies spinal decompressions among other uses. To improve the rate of fusion if local bone is insufficient, surgeons often use graft material. Autologous iliac crest bone graft (ICBG) is considering the gold standard for grafting but often involves harvesting bone from a separate site through a separate incision.

Recombinant bone morphogenetic protein-2 (rhBMP-2) is an orthobiologic that promotes bone formation and spinal fusion that is used as an alternative to ICBG. It is approved for anterior lumbar interbody fusion (ALIF) surgery but was widely used in other surgical approaches with off-label use estimated at 85% [36] and peak sales approach $1 billion [37].

Adverse events attributed to rhBMP-2 began to be reported. In 2008, the US Food and Drug Administration (FDA) issued a warning about dangerous side effects associated with rhBMP-2 use in anterior cervical spine surgery including dysphagia, hematoma, seroma, swelling, and the need for intubation. Subsequent studies brought attention to unexpected adverse events in surgical approaches outside the approved anterior lumbar approach including radiculitus, vertebral body resorption, seroma and/or hematoma formation, and heterotopic ossification. Even in the approved ALIF approach, there were reported associated with osteolysis and retrograde ejaculation [38]. A review of publicly available data in 2011 sparked the controversy which led to this systematic review effort asserting an underreporting of adverse events including a risk of cancer [39].

## Abbreviations

AHRQ: Agency for Healthcare Research and Quality
ALIF: Anterior lumbar interbody fusion
ICBG: Iliac crest bone graft
IPD: Individual participant-level data
OHSU: Oregon Health & Science University
PLF: Posterolateral fusion
RCT: Randomized controlled trials
rhBMP-2: Recombinant human bone morphogenetic protein-2
YODA: Yale University Open Data Access
York: University of York
